# Applications and Engineering Analysis of Lotus Roots under External Water Pressure

**DOI:** 10.1155/2016/2386982

**Published:** 2016-12-29

**Authors:** Yiyun Zhu, Chang Jiang Wang, Diane Mynors

**Affiliations:** ^1^School of Civil Engineering and Architecture, Xian University of Technology, No. 5 Jinhua South Road, Xian, Shaanxi 710048, China; ^2^School of Engineering and Informatics, University of Sussex, Brighton BN1 9QJ, UK

## Abstract

Engineers can learn from nature for inspirations to create new designs. The internal structure of lotus roots with several oval holes was studied in this paper for engineering inspirations. The structural performance of lotus roots under outside water pressure was simulated and compared with various cross-sectional areas. The distribution of stresses in the cross-sectional area of lotus roots was analysed and presented. It was found that the maximum compressive stresses in the cross-sectional area of lotus roots were occurring at the long axis ends of the holes. This was very different from that of circular holes. Further analysis on the triaxiality factors revealed that the cross-sectional area of the lotus root resulted in large areas of high triaxiality factors. The resulting hydrostatic stress in the cross-sectional area of lotus root ranges from zero to 2.7 times the applied outside pressure. In contrast, the hydrostatic stress in a cylindrical cross-sectional area is a fixed value. The study showed that the lotus root and the orientation of the oval holes could be mimicked in the design of new structures, for example, underwater pipes and vessels.

## 1. Introduction

Through evolution, nature has learned to achieve maximal performance by using minimum resources. It has evolved and optimized a large number of materials and structured surfaces with rather unique characteristics [1]. Therefore, adopting designs based on the study of plants and animals in the field of biomimetics or bionics is important as biological systems produce many functions that can be applied in engineering; many examples have been presented and discussed by Vincent [2]. The benefits gained from biomimetics are not totally obvious; therefore, the practical use of mechanisms of functions in engineering and other disciplines is still young [3]. The biological system should be studied and understood before the ideas from biology can be transferred into engineering and design.

Structural optimization is very important in the design of mechanical systems in industry. Shape optimization of engineering components can follow the design rules of nature; for example, Mattheck [4] studied the tree fork and observed that trees can maintain a uniform stress distribution at their surface through load-adaptive growth. Mattheck [4] then proposed a method of tensile triangles to remove unloaded parts within a structure in order to save materials.

In this paper, lotus roots with large and small holes under external water pressures will be studied to extract nature's design principles. Lotus roots are found buried in anaerobic sediment and are characterised by having oval holes for obtaining oxygen. Mevi-schutz and Grosse [5] conducted experiments that showed that thermoosmotic gas transport could drive oxygen flow from the lotus leaves to the roots. Mevi-schutz and Grosse [6] also showed a lacunar pressure of up to 166 ± 44 Pa that could be measured in both young and old lotus leaves. The standard atmospheric pressure is 101325 Pa; therefore, it can be reasonably assumed that the gas pressures inside the lotus root holes are close to the atmospheric pressure when the structural analysis was conducted in this paper.

Dominy et al. [7] have studied the mechanical properties of plant underground storage organs. They found that rhizomes were the most resistant to deformation and fracture, followed by tubers, corms, and bulbs. They used a portable universal tester to estimate Young's modulus and fracture toughness of a range of plant species, with Young's modulus varying between 0.8 MPa and 18.7 MPa.

Vincent [8] reported many advantages of using holes in engineering structures, for example, making an object lighter and more durable, and holes also can affect the way that a material fails. It was pointed out by Vincent [8] that engineers and designers treat holes with suspicion and are not using their advantages because we do not always know how best to use them.

The study of the effect of holes on the strain distribution in Campaniform Sensilla by Vincent et al. [9] showed that the orientation of the hole with respect to the applied load is significant, and the effects of grouping and mutual proximity of the holes are important in strain magnification as well.

The lotus root has a unique geometry with its canals regularly aligned. Through the study of the lotus root's porosity and orderly arranged pores, the lotus root has already provided engineering inspirations for the designs of a multibore hollow fibre membrane [10] and a porous nanocomposite polymer electrolyte [11]. It has also been applied to the development of porous carbon steels [12].

Chen and Zhang [13] reported that the enlargement of parenchymatous cells resulted in the growth or thickening of the rhizome. Niklas [14] reported that tissue composite modulus should be named for the elastic modulus obtained from mechanical test, because it is different from the modulus for solid materials. The elastic modulus of parenchyma tissue is reported to be between 3 MPa and 6 MPa; the compressive strength is between 0.27 MPa and 1.3 MPa [15]. Stresses will be developed in the lotus roots when outside water/mud loads are applied; these internal stress states can affect cell expansion. To analyse the state of stress in lotus roots, triaxiality and hydrostatic stress will be discussed.

Material properties can be affected by hydrostatic stress in material deformations. Triaxiality is mainly used to describe the forming limit of materials and ductile fracture criteria. The triaxiality factor (TF) in a material is a ratio of the hydrostatic stress and the von Mises stress resulted from loading. Bridgman [16] reported that hydrostatic stress could increase the ductility of metals but not result in plastic deformation. With certain hydrostatic stress levels, brittle materials can be deformed like ductile materials. In uniaxial tension, the TF is 1/3; in uniaxial compression, TF is −1/3; and, in biaxial tension, TF is 2/3. When compressive principal stresses occurred in materials, TF is less than 1/3. Kweon [17] studied material damage and showed that no shear damage can occur when the TF is smaller than −1/3. Bao and Wierzbicki [18] showed that fracture never occurs when the stress triaxiality value is smaller than −1/3.

The novelty of this paper is to analyse and reveal the effects of the layout and orientation of the large and small holes on the stress distribution in the cross-sectional area of lotus roots through finite element simulations. To this end, the hydrostatic stress and triaxiality in the cross-sectional area of lotus with oval holes will be analysed and compared with the cross-sectional areas of various artificial arrangements of holes. From this, the natural design principles of the lotus root is extracted and applied to solve an engineering problem as a proof of concept.

## 2. Methods

### 2.1. The Structure of Lotus Roots

**Figure 1:**
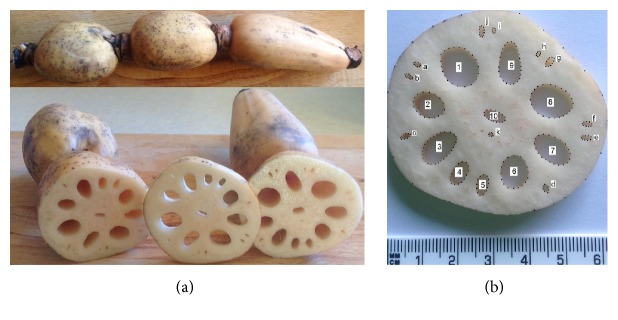
(a) Lotus roots and (b) a slice of lotus root used for the analysis.

The fresh lotus root shown in Figure 1(a) was bought from a supermarket and the origin is not known, only the dimensions of the cross-sectional area are important in this research. The lotus root was cut at the middle section using a kitchen knife and the slice used for study is shown in Figure 1(b). The slice dimensions were measured using the pixels alongside the scale of the picture in Figure 1(b), and the holes were numbered and labelled for 2D finite element modelling and engineering analysis.

The area of each hole in the lotus root shown in Figure 1(b) is included in Table 1. The solid area of the cross-sectional area of the lotus root in Figure 1(b) is 2138 mm^2^. The total area of large holes numbered from 1 to 10 is 506 mm^2^, with a mean value of 50.6 mm^2^ and a standard deviation of 26.7 mm^2^. The total area of the small holes labelled alphabetically from a to k is 28 mm^2^, with a mean value of 2.5 mm^2^ and a standard deviation of 1.1 mm^2^. The total area of all the holes and the solid area is 2672 mm^2^. Therefore, the porosity of the cross-sectional area of the lotus root is 20%.

### 2.2. Finite Element Model of the Cross-Sectional Area of Lotus Root

**Figure 2:**
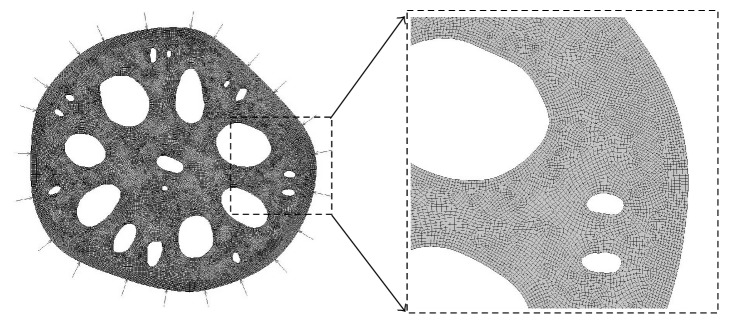
Finite element model of the cross-sectional area of lotus root.

ANSYS [19] finite element software was used for the simulation. The finite element model of the cross-sectional area of the lotus root and the meshes are shown in Figure 2. The element size of 0.2 mm is used after convergence testing; the finite element model consists of 46733 2D quadrilateral elements. A plane strain analysis is used for the stress analysis and finite element modelling. The specific lotus root used in this research was not mechanically tested, as it is difficult to characterise the mechanical properties of biomaterials. However, for comparative analysis, assumptions from prior studies can be reasonably used: an elastic modulus of 5 MPa is adopted from the work by Gibson [15] and Poisson's ratio of 0.3 was used from the work by Wei et al. [20] and Hamza et al. [21]. An outside pressure of 0.01 MPa was used to simulate a water depth of 1 metre, and this pressure is applied to the external boundary of the cross-sectional area of lotus root. Zero displacements are applied at a node on the centre hole of section to provide numerical stability during solving process.

### 2.3. Stresses in the Thick-Walled Cylinder

**Figure 3:**
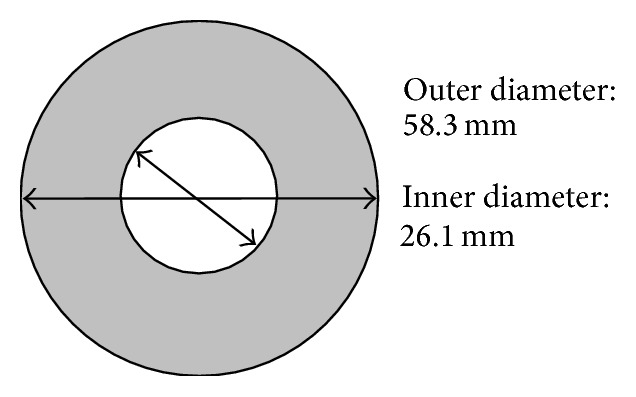
A cylindrical cross section of the same area and porosity of the lotus root.

When the cross-sectional area of the lotus root is equivalently represented as a cylindrical cross section as shown in Figure 3, the outer and inner diameters of the hollow circular cross section are 58.3 mm and 26.1 mm in order to maintain the same values of porosity and area. Using Lamé's equation (1) for thick-walled cylinders, the tangential compressive stress on the inner surface of the hollow circular cross-sectional area is 0.025 MPa when the area is subject to an outside water pressure of 0.01 MPa:

(1) $$\begin{array}{c} \sigma_{\theta }=\frac{-P_{o}r_{o}^{2}}{r_{o}^{2}-r_{i}^{2}}\left(\;1+\frac{r_{i}^{2}}{r^{2}}\;\right). \end{array}$$

## 3. Results and Discussion

### 3.1. Compressive Stresses in the Cross-Sectional Area of the Lotus Root

**Figure 4:**
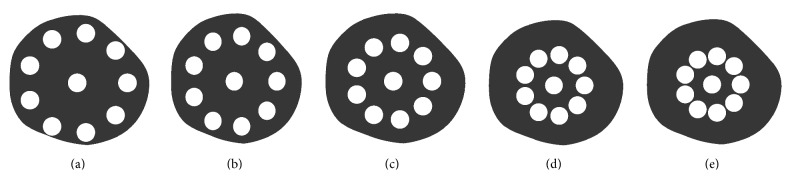
Circular holes in the cross-sectional area of the lotus root.

To understand the influence of hole shape, the actual holes in the cross-sectional area were replaced with 10 circular holes for comparison. Each circular hole has a diameter of 4.12 mm to create the same porosity in the modified cross sections when compared to the actual lotus root. One circular hole is located at the centre of the lotus root cross section and the other nine circular holes are equally distributed along the circumference of a “planetary” circle with its centre coinciding with the centre of the lotus root. The locations of the nine outer circular holes are varied by changing the diameter of the planetary circle and the effects on stress distributions are studied. The diameter of the planetary circle is varied from 22 mm, 20 mm, 17 mm, and 14 mm to 13 mm. These five cases are labelled as (a) to (e) as shown in Figures 4(a)to 4(e) respectively.

**Figure 5:**
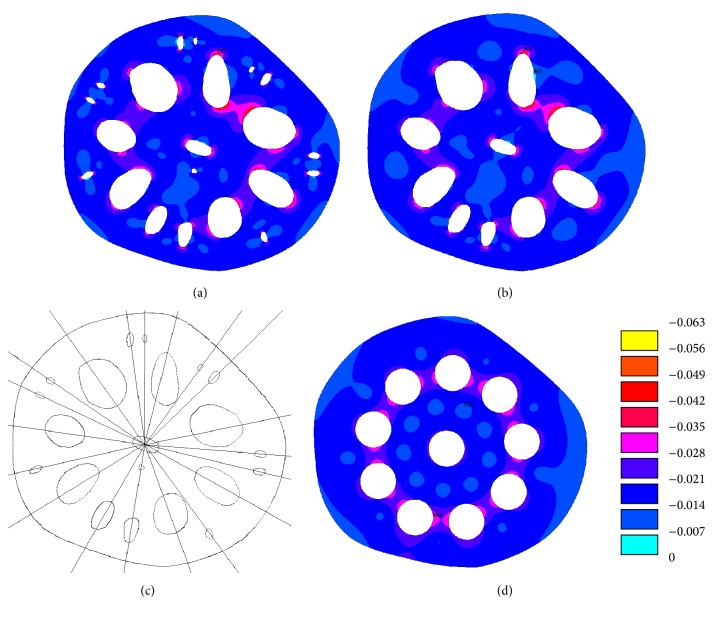
Compressive stress (MPa) in the lotus root (a) with small holes, (b) no small holes, (c) radial lines through oval holes, and (d) compressive stress in the cross-sectional area with circular holes.

In Figure 5(a), the highest compressive stresses were developed in the area of small holes. It was noted that the maximum compressive stress in the cross-sectional area of the lotus root with smaller holes (case 1) was 0.058 MPa and 0.060 MPa in the cross-sectional area without smaller holes (case 2). These compressive stresses are also greater than that in the cylindrical cross-sectional area shown in Figure 3. Comparing the stresses in Figures 5(a) and 5(b), the maximum compressive stress in the lotus root with the small holes was reduced by 0.002 MPa, yielding a reduction of 3%. These small holes reduce the stress in the cross-sectional area of lotus root and therefore they should be included in the structural analysis.

From the compressive stress plot in Figure 5(a), it can be seen that the compressive stresses are highest at the two major axis ends of the oval holes. It was noticed that the compressive stresses are located mostly at where the oval holes intersect with the radial lines that are shown in Figure 5(c) in the cross-sectional area of lotus root. However, the compressive stress distributions around circular holes are not the same as the oval holes. The compressive stresses are located near where the circular holes intersect with the planetary circle, as shown in Figure 5(d).

### 3.2. Tensile Stresses in the Cross-Sectional Area of the Lotus Root

**Figure 6:**
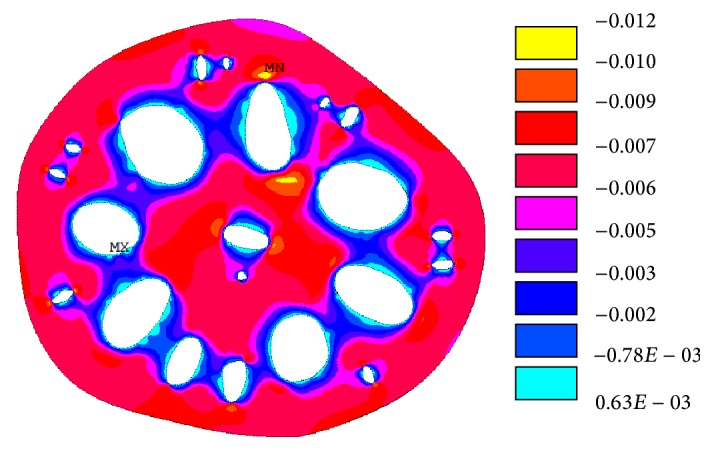
Tensile stress (MPa) distribution in the cross-sectional area of the lotus root.

Tensile stresses in the cross-sectional area of the lotus root are presented in Figure 6 where the maximum tensile stress value is 0.00063 MPa, which is about 1% of the maximum compressive stress in the lotus root. The tensile stresses could be close to zero if the surfaces of the holes in the finite element model were a true representation of the natural lotus root.

### 3.3. The von Mises Stresses in the Cross-Sectional Area of the Lotus Root

The von Mises criterion or maximum distortion energy criterion is associated with the change in shape of the material and is normally applied to ductile materials like metals. However, because lotus roots are subjected to water pressure acting on the external surfaces, it is considered useful that the von Mises stress is discussed in this paper.

**Figure 7:**
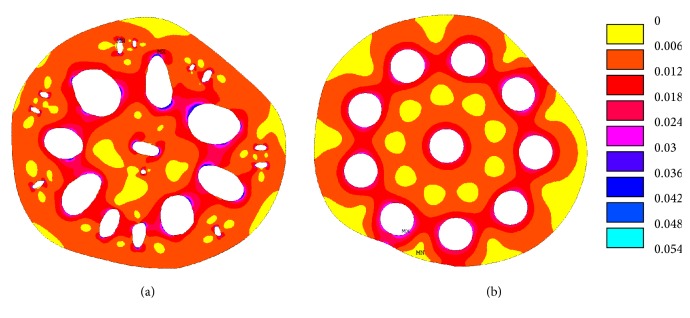
The von Mises stress (MPa) distribution: (a) the actual lotus root and (b) lotus root with circular holes.

The von Mises stress distributions in the actual cross-sectional area of the lotus root (case 1) and with circular holes (case c) are shown in Figure 7. It was noticed that higher stresses were developed around the holes in the actual cross-sectional area of the lotus root; it was also calculated that the total area of stresses between 0 MPa and 0.006 MPa in the actual lotus root cross section is smaller than that of case c.

**Figure 8:**
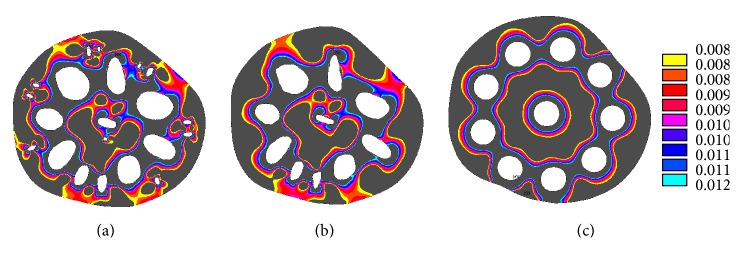
The von Mises stress (MPa) in the cross-sectional area of lotus root (a) with small holes, (b) no small holes, and (c) with circular holes.

In order to compare the von Mises stress distribution, von Mises stresses between 0.008 MPa and 0.012 MPa were studied, that is, 80% to 120% of the applied outside pressure. These stresses are shown in Figure 8 and it is noted that these stresses are distributed close to the holes. In order to show the effect of the distribution of the holes on the stress values and stressed area, the area of the von Mises stresses between 0.008 MPa and 0.012 MPa in each case is listed in Table 2. In case 1, the total area of having a von Mises stress between 0.008 MPa and 0.012 MPa is 691 mm^2^, which is 32.3% of the solid area of the cross-sectional area of the lotus root. Comparing case 1 and case 2 in Table 2, when the small holes were considered in the modelling of the lotus root, nearly more than 10% of the areas are in stress states between 0.008 MPa and 0.012 MPa. Comparing case 1 with cases a, b, and c, it was noticed that the circular holes in cases a, b, and c resulted in a smaller area, being in the stress states between 0.008 MPa and 0.012 MPa. Cases d and e showed a larger area, being in the stress states between 0.008 MPa and 0.012 MPa. However, cases d and e are not truly representative of actual lotus root because these circular holes are very close to each other and little material is left between the holes, weakening the structure. The extreme cases of d and e showed that the lotus root should be correctly mimicked in engineering applications.

### 3.4. Triaxiality Factors in the Cross-Sectional Area of Lotus Root

Influence of holes on the triaxiality factors in the cross-sectional area shown in Figure 2 will be compared with the cylindrical cross-sectional area shown in Figure 3. The theoretical triaxiality factors in the cylindrical cross-sectional area will be calculated and used for analysis.

**Figure 9:**
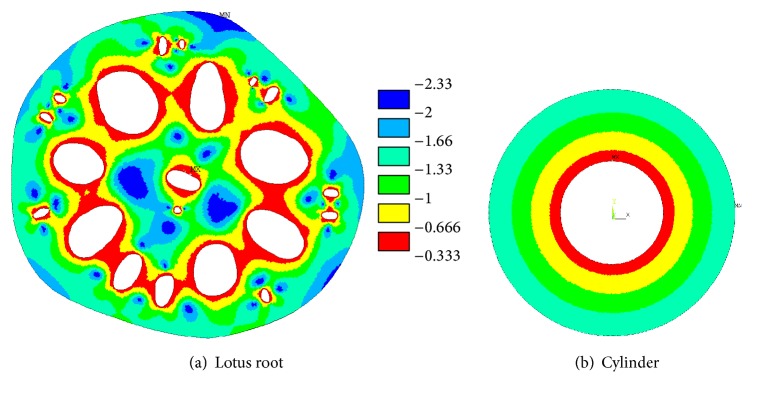
Triaxiality factors in the cross-sectional areas.

With an outside water pressure load of 0.01 MPa, the triaxiality factors in the cross-sectional area of the lotus root and the cylindrical cross-sectional area is shown in Figure 9. It can be seen that high triaxiality factors are at the vicinity of these holes. For a cylindrical cross-sectional area, the triaxiality factors under plane strain assumption is shown by (2), where *r*, *r*_*i*_, and *ν* are the outside, inside radius and Poisson's ratio, respectively:

(2) $$\begin{array}{c} TF=\frac{2\left(1+\nu \right)}{\sqrt[{3}]{3\left(r_{i}^{4}/r^{4}\right)+{\left(1-2\nu \right)}^{2}}}. \end{array}$$

Taking *ν* as 0.3, since *r*_*i*_/*r* ≈ 1, for a thin walled circular cross-sectional area, then TF = −0.49, when *r*_*i*_ = 0, TF = −2.17.

In order to show the difference between triaxiality factors in the cross-sectional areas shown in Figure 9, the area of each triaxiality factor group is calculated and shown in Table 3. Elements of triaxiality factors from −1/3 to −2/3 are collected as group 1, which represents the TF value close to a thin walled cylindrical cross-sectional area. Elements of triaxiality factors from −2 to −7/3 are collected as group 6, which represents the value of a solid circular cross-sectional area. From the analysis of triaxiality factors in the cross-sectional area of lotus root and hollow cylinders, there are large amounts of areas with triaxiality factors between −1/3 and −2/3 in the cross-sectional area of the lotus root compared to that of cylindrical cross-sectional area, that is, showing the behaviour of a thin walled section when the lotus root is under outside water pressure.

### 3.5. Hydrostatic Stress to External Pressure Factor (HPF)

**Figure 10:**
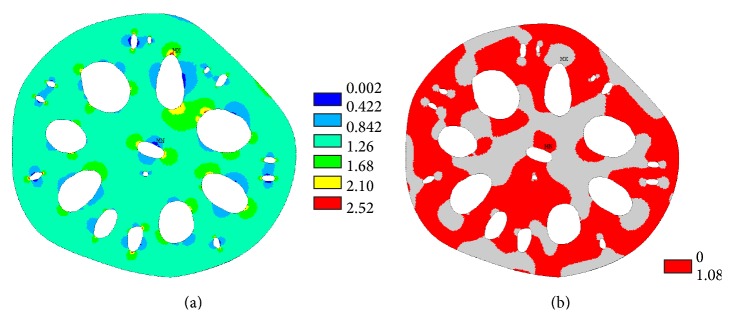
(a) HPF in the cross-sectional area of the lotus root and (b) HPF values less than 1.08.

To compare and analyse hydrostatic stress, an internal hydrostatic stress pressure factor (HPF) is introduced here; it is the hydrostatic stress developed in the cross-sectional area divided by the applied outside pressure. For example, the HPF in the cylindrical cross-sectional area shown in Figure 3 is calculated as 1.08, while the HPF in a solid circular cross-sectional area is 0.87. Analysing the finite element numerical results, the HPF from 0.02 to 2.52 in the cross-sectional area of lotus root is shown in Figure 10(a); this is different from the cylindrical cross-sectional area that has a constant HPF value. In Figure 10(b), the HPF in the majority of the cross-sectional area of the lotus root is smaller than 1.08, and higher HPF values occur near the major axis ends of the holes in the lotus root.

### 3.6. Engineering Applications

**Figure 11:**
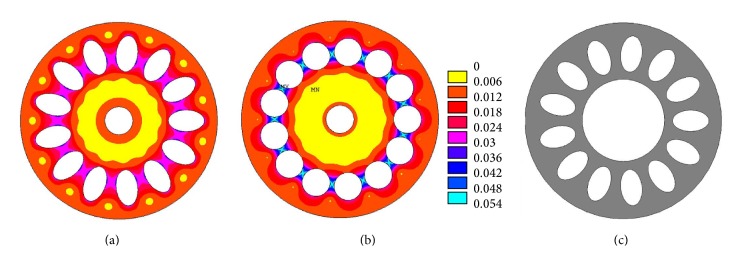
(a) The von Mises stress (MPa) in a section with oval holes, (b) von Mises stress in a section with circular holes, and (c) a proposed pipe cross section.

Lotus roots are normally subjected to outside water pressures, and the formation of oval/elliptical holes in cross-sectional area is interesting to engineers; this research work can provide an inspiration for design ideas of underwater vessels and other structures having several compartments. To illustrate the advantage of using oval holes like that in the lotus roots, von Mises stress distributions of two cross-sectional areas are shown in Figures 11(a) and 11(b). The two cross-sectional areas have the same porosity, solid area, outer diameter, and number of holes. The von Mises stress in the section of circular holes is about 60% higher than that in the section of oval holes subject to an outside pressure of 0.01 MPa. A challenging problem in exploiting oil resources from deep seas in the petroleum industry is that the transportation pipes are subjected to high hydrostatic pressure and low temperatures. Bouchonneau et al. [22] tested an insulated deep sea pipe structure composed of a steel pipe of internal diameter 180 mm, wall thickness 12 mm, and a 5-layer insulating coating which is mainly polypropylene with a total thickness of 61 mm. Based on these dimensions and from the stress distributions shown in Figures 11(a) and 11(b), a pipe as shown in Figure 11(c) could be designed and used in the deep sea. This pipe with oval holes could be made through the extrusion of materials. The results in this research support the findings of Vincent [8] that if holes are sensibly placed and have the right dimensions, they can improve the durability of a material or structure. The hydrostatic stress and triaxiality in the cross-sectional area of the lotus roots and cross-sectional area of the pipe are different when they are under external pressure. The lotus root could inspire engineers to create new materials and novel structures.

## 4. Conclusions

Because of the oval holes in lotus roots, large compressive stresses in the cross-sectional area of the lotus root occurred at the major axis ends of the actual holes. When the oval holes were simulated as circular holes, the maximum compressive stress locations were changed to where the short axis of the oval holes was located. Under the outside water pressure, the oval holes in the cross-sectional area of the lotus roots resulted in more areas having the von Mises stress close to the outside water pressure than the circular holes. Very small or negligible tensile stresses occurred in the cross-sectional areas of lotus roots under outside water pressures.

Under the outside water pressure, the cross-sectional area of the lotus root showed that the hydrostatic stress is varying. This is different from the constant hydrostatic stress values in the cross-sectional area of cylinder. The analysis also showed that large areas of high triaxiality factors occurred in the cross-sectional area of the lotus root, and all the triaxiality factors are negative. Because the shape of holes in the lotus root can change the stress states, the creation of new materials and structures can be inspired by lotus roots.

## Figures and Tables

**Table 1:** Area of holes in the cross-sectional area of the lotus root.

Large hole	1	2	3	4	5	6	7	8	9	10	
Area (mm^2^)	90.8	48.8	58.5	21.9	14.4	55.6	59.8	85.1	56.6	14.9	

Small hole	a	b	c	d	e	f	g	h	i	j	k
Area (mm^2^)	2.1	2.1	3.2	2.8	3.0	2.9	4.5	1.3	1.2	3.8	1.1

**Table 2:** Area in the lotus root cross section with von Mises stresses between 0.008 MPa and 0.012 MPa.

	Case 1	Case 2	Case a	Case b	Case c	Case d	Case e
Area (mm^2^)	691	641	569	471	532	908	1013

**Table 3:** Area of groups of triaxiality factor (mm^2^).

Group of triaxiality factor	1	2	3	4	5	6
Triaxiality factor	−1/3 to −2/3	−2/3 to −1	−1 to −4/3	−4/3 to −5/3	−5/3 to −2	−2 to −7/3
Lotus root section	330	443	440	586	261	79
Cylindrical section	214	456	604	861	0	0

## Abbreviations

TF: Triaxiality factor
HPF: Hydrostatic stress to external pressure factor.
